# Evidence to support natural hybridization between *Anopheles sinensis* and *Anopheles kleini* (Diptera: Culicidae): possibly a significant mechanism for gene introgression in sympatric populations

**DOI:** 10.1186/1756-3305-7-36

**Published:** 2014-01-20

**Authors:** Wej Choochote, Gi-Sik Min, Pewpan M Intapan, Chairat Tantrawatpan, Atiporn Saeung, Viraphong Lulitanond

**Affiliations:** 1 Department of Parasitology, Faculty of Medicine, Chiang Mai University, Chiang Mai 50200, Thailand; 2 Department of Biological Sciences, Inha University, Incheon 402-751, South Korea; 3 Research and Diagnostic Center for Emerging Infectious Diseases, Khon Kaen University, Khon Kaen 40002, Thailand; 4 Department of Parasitology, Faculty of Medicine, Khon Kaen University, Khon Kaen 40002, Thailand; 5 Division of Cell Biology, Department of Preclinical Sciences, Faculty of Medicine, Thammasat University, Rangsit Campus, Pathum Thani 12121, Thailand; 6 Department of Microbiology, Faculty of Medicine, Khon Kaen University, Khon Kaen 40002, Thailand

**Keywords:** *Anopheles sinensis*, *An. kleini*, Hybridization experiment, Second internal transcribed spacer, Cytochrome *c* oxidase subunit I, Introgression

## Abstract

**Background:**

Malaria caused by *Plasmodium vivax* is still a public health problem in the Republic of Korea (ROK), particularly regarding the recent re-emergence of this malarial species near the demilitarized zone in northwestern Paju City, Gyeonggi-do Province. Currently, at least 4 species (*An. kleini*, *An. pullus*, *An. belenrae* and *An. lesteri*) of the Hyrcanus Group are reported as possible natural vectors of vivax malaria in the ROK, and *An. sinensis*, which is the most dominant species, has long been incriminated as an important natural vector of this *P. vivax*. However, *An. sinensis* was ranked recently as a low potential vector. According to the discovery of natural hybrids between *An. sinensis* (a low potential vector for *P. vivax*) and *An. kleini* (a high potential vector for *P. vivax*) in Paju City, intensive investigation of this phenomenon is warranted under laboratory conditions.

**Methods:**

Mosquitoes were collected during 2010-2012 from Paju City, ROK. Hybridization experiments used iso-female line colonies of these anophelines together with DNA analysis of ribosomal DNA [second internal transcribed spacer (ITS2)] and mitochondrial DNA [cytochrome *c* oxidase subunit I (COI)] of the parental colonies, F_1_-hybrids and repeated backcross progenies were performed intensively by using a PCR-based assay and pyrosequencing technology.

**Results:**

The results from hybridization experiments and molecular investigations revealed that the mitochondrial COI gene was introgressed from *An. sinensis* into *An. kleini*. The *An. sinensis* progenies obtained from consecutive repeated backcrosses in both directions, i.e., F_2_-_11_ progeny [(*An. sinensis* x *An. kleini*) x *An. sinensis*] and F_3_-_5_ progeny [(*An. kleini* x *An. sinensis*) x *An. kleini*] provided good supportive evidence.

**Conclusions:**

This study revealed introgression of the mitochondrial COI gene between *An. sinensis* and *An. kleini* through consecutive repeated backcrosses under laboratory conditions. This new body of knowledge will be emphasized in reliable promising strategies in order to replace the population of *An. kleini* as a high potential vector for *P. vivax*, with that of a low potential vector, *An. sinensis*, through the mechanism of gene introgression in nature.

## Background

Up until now, at least 26 species members of the *Anopheles hyrcanus* group have been reported, and their distribution has extended widely from Europe to East and Southeast Asia, including some of the off-lying islands of the Indian and Pacific Oceans [1]. Some species of the Hyrcanus Group are accepted as important vectors in transmitting human diseases, e.g., malaria (*Plasmodium vivax*) [2-14], filariae (*Wuchereria bancrofti* and *Brugia malayi*) [15,16], and Japanese encephalitis virus [17,18], particularly in the Oriental and contiguous parts of eastern Palaearctic regions.

At least 6 species (*Anopheles sinensis*, *An. lesteri*, *An. pullus*, *An. sineroides*, *An. belenrae* and *An. kleini*) of the Hyrcanus Group are found indigenously in the Republic of Korea (ROK). Among these, *An. sinensis* has long been incriminated as the most dominant and important natural vector of *P. vivax*, especially due to the recent re-emergence of vivax malaria near the demilitarized zone in northwestern Paju City, Gyeonggi-do Province [3,19-22]. However, the low concentration of circumsporozoite (CS) antigen obtained from wild-caught females [6], and very low sporozoite rates recovered from laboratory susceptibility tests [7,9], have brought about the cryptic status of *An. sinensis* as a natural vector of vivax malaria transmission in the ROK. Consequently, the implication of other *An. hyrcanus* species, i.e., *An. kleini*, *An. pullus*, *An. belenrae* and *An. lesteri* as possible natural vectors of vivax malaria in the ROK has been proposed extensively [8,9], even though the latter species is thought to have a small population [7]. Remarkably, *An. sinensis* strain from China has been incriminated recently as an efficient vector of *P. vivax*[11].

The discovery of natural hybrids has been reported from some important malaria vectors, for instance, between *An. gambiae* and *An. arabiensis*[23], *An. scanloni* (= *dirus* C) and *An. baimaii* (= *dirus* D) [24], and *An. minimus* and *An. harrisoni*[25,26]. Regarding *An. kleini* (a high potential vector for *P. vivax*) and *An. sinensis* (a low potential vector for *P. vivax*) [7,9], a single gravid, natural hybrid female was discovered between these 2 anopheline species from a total of 658 wild-caught females in Paju City, ROK; an endemic zone of vivax malaria from 2004 to 2008. These wild-caught females comprised 360 *An. sinensis*, 258 *An. pullus*, 20 *An. belenrae*, 15 *An. kleini*, 3 *An. sineroides*, 1 *An. lesteri* and 1 hybrid female between *An. kleini* and *An. sinensis*[27]. The results of self-crossing between F_1_-progenies derived from one egg-batch of an egg-laid gravid female, and molecular investigations, revealed possible natural backcrossing (introgression) between a hybrid female and male *An. sinensis*. There is no clear basic information on the introgression between *An. sinensis* and *An. kleini*, as proposed by [27]. Thus, systematic investigations into the role of introgressive hybridization between these two anopheline species were performed in this study. Accordingly, attempts were made to establish iso-female line colonies of *An. sinensis* and *An. kleini*, perform crossing experiments (reciprocal and back crosses), investigations of reproductive systems of hybrid and backcross progenies, and compare DNA analysis of ribosomal DNA (ITS2) and mitochondrial DNA (COI) of the parental, F_1_-hybrids with that of repeated backcross progenies by using a PCR-based assay and pyrosequencing technology.

## Methods

### Establishment of iso-female lines

Mosquitoes were collected during 2010-2012 from Paju City, Republic of Korea (ROK), by placing a light trap in cowsheds. Then, wild-caught females were transported for colonization to the insectary of the Department of Parasitology, Faculty of Medicine, Chiang Mai University, Chiang Mai, Thailand. Four iso-female lines of both *An. sinensis* and *An. kleini* were established successfully using the methods of [28]. An F_1_-progeny of each iso-female line was used for species identification following the keys of [29] as well as a molecular assay [30]. Then, one iso-female line of each species, with molecular identification of both nuclear (ITS2) and mitochondrial (COI) genes, were well matched with those in the GenBank nucleotide sequence database, and selected, i.e., *An. sinensis* F_0_-1 (SF0-1) and *An. kleini* F_0_-1 (KF0-1). These iso-female lines have been maintained in colonies in the laboratory at Chiang Mai University for more than 10 consecutive generations, and used for hybridization experiments and comparative DNA sequence analyses.

### Hybridization experiments

Hybridization experiments (reciprocal and back crosses, and repeated backcross progenies) between *An. sinensis* and *An. kleini* were performed by using virgin females and males and following the techniques previously reported by [31]. Post-mating reproductive isolation was recorded using the criteria of low viability (hatchability, survival, pupation, and emergence), adult sex distortion and abnormal morphology of the reproductive system.

### PCR identification, dideoxy sequencing and phylogenetic analysis

DNA was extracted individually from 60 mosquitoes using the RED Extract-N-Amp™ Tissue PCR kit (Sigma-Aldrich, Spruce Street, SL) as shown in Table 1. Primers for the amplification of ITS2 and COI regions followed a previous report by [30]. The ITS2 region of the rDNA was amplified using the ITS2 Forward and ANO 28S-20 primers [30,32]. The mitochondrial COI gene was amplified using the LCO1490 (5′-GGT CAA CAA ATC ATA AAG ATA TTG G-3′) and HCO2198 (5′-TAA ACT TCA GGG TGA CCA AAA AAT CA-3′) primers of [33]. PCR reaction was carried out in a total volume of 25 μl containing 10 pM of each primer; and 2.5 μl of 10X buffer containing 50 mM KCl, 10 mM Tris-HCI, 0.1% Triton^®^X 100 supplemented with 1.5 mM MgCl_2_ (Promega, USA), 200 μM of each dNTP (GeneCraft, Germany), 0.5 μl of *Taq* DNA polymerase (Promega, USA) and 10-100 pg of genomic DNA. The amplification profile comprised initial denaturation at 94°C for 3 min, with 30 cycles at 94°C for 30 sec, 55°C for 30 sec, and 72°C for 2 min, and a final extension at 72°C for 7 min. The PCR products were separated by electrophoresis on a 1.5% agarose gel stained with ethidium bromide. Finally, the purified PCR products were subjected to sequencing in an ABI PRISM 3700 DNA Analyzer (Applied Biosystems, Foster City, CA) using a Dye Terminator Cycle Sequencing Ready Reaction Kit (Applied Biosystems). The sequence data obtained were deposited in the GenBank nucleotide sequence database (Table 1). The newly determined ITS2 and COI sequences were also compared with those available in GenBank, using the Basic Local Alignment Search Tool (BLAST) available at http://blast.ncbi.nlm.nih.gov/Blast.cgi. The DNA sequence data were edited manually in BioEdit version 7.0.5.3 [34] and aligned using CLUSTAL W [35]. Constructions of neighbor-joining trees using distance [36], and the bootstrap test with 1,000 replications, were performed with the MEGA version 4.0 program based on COI sequences [37]. The COI sequences of *An. peditaeniatus* (GenBank accession number AB539069) [38] and *An. pullus* (GenBank accession number AB444348) [39] were included in phylogenetic analysis.

**Table 1:** **Species identification of *An. sinensis *** **and *An. kleini *** **samples based on ITS2 and COI sequences, and their GenBank accession numbers**

**Samples (Female x Male)**	**Code of samples**	**Identified species name (GenBank accession number)**		**Reference**
		**ITS2**	**COI**	
Parental				
*An. sinensis* F_0_-1	SF0-1	*An. sinensis (KC797396)*	*An. sinensis (KC797435)*	This study
*An. sinensis* F_0_-2	SF0-2	*An. sinensis (KC797397)*	*An. sinensis (KC797436)*	This study
*An. sinensis* F_0_-3	SF0-3	*An. sinensis (KC797398)*	*An. sinensis (KC797437)*	This study
*An. sinensis* F_0_-4	SF0-4	*An. sinensis (KC797399)*	*An. sinensis (KC797438)*	This study
*An. kleini* F_0_-1	KF0-1	*An. kleini (KC797431)*	*An. kleini (KC797439)*	This study
*An. kleini* F_0_-2	KF0-2	*An. kleini (KC797432)*	*An. sinensis (KC797440)*	This study
*An. kleini* F_0_-3	KF0-3	*An. kleini (KC797433)*	*An. sinensis (KC797441)*	This study
*An. kleini* F_0_-4	KF0-4	*An. kleini (KC797434)*	*An. sinensis (KC797442)*	This study
Reciprocal crosses				
*An. sinensis* x *An.kleini* F_1_-1	SKF1-1	Mixed	*An. sinensis (KC797446)*	This study
*An. sinensis* x *An.kleini* F_1_-2	SKF1-2	Mixed	*An. sinensis (KC797447)*	This study
*An. sinensis* x *An.kleini* F_1_-3	SKF1-3	Mixed	*An. sinensis (KC797448)*	This study
*An. kleini* x *An. sinensis* F_1_-1	KSF1-1	Mixed	*An. sinensis (KC797443)*	This study
*An. kleini* x *An. sinensis* F_1_-2	KSF1-2	Mixed	*An. kleini (KC797444)*	This study
*An. kleini* x *An. sinensis* F_1_-3	KSF1-3	Mixed	*An. sinensis (KC797445)*	This study
Back crosses				
(*An. sinensis* x *An. kleini*) x *An. sinensis*				
hybridF_1_ x *sinensis*-1 = > (hybridF_2_-1)	SKSF2-1	Mixed	*An. sinensis (KC797449)*	This study
hybridF_1_ x *sinensis*-2 = > (hybridF_2_-2)	SKSF2-2	Mixed	*An. sinensis (KC797450)*	This study
hybridF_1_ x *sinensis*-3 = > (hybridF_2_-3)	SKSF2-3	Mixed	*An. sinensis (KC797451)*	This study
hybridF_2_ x *sinensis*-1 = > (hybridF_3_-1)	SKSF3-1	*An. sinensis (KC797400)*	*An. sinensis (KC797452)*	This study
hybridF_2_ x *sinensis*-2 = > (hybridF_3_-2)	SKSF3-2	*An. sinensis (KC797401)*	*An. sinensis (KC797453)*	This study
hybridF_2_ x *sinensis*-3 = > (hybridF_3_-3)	SKSF3-3	*An. sinensis (KC797402)*	*An. sinensis (KC797454)*	This study
hybridF_3_ x *sinensis*-1 = > (hybridF_4_-1)	SKSF4-1	*An. sinensis (KC797403)*	*An. sinensis (KC797455)*	This study
hybridF_3_ x *sinensis*-2 = > (hybridF_4_-2)	SKSF4-2	*An. sinensis (KC797404)*	*An. sinensis (KC797456)*	This study
hybridF_3_ x *sinensis*-3 = > (hybridF_4_-3)	SKSF4-3	*An. sinensis (KC797405)*	*An. sinensis (KC797457)*	This study
hybridF_4_ x *sinensis*-1 = > (hybridF_5_-1)	SKSF5-1	*An. sinensis (KC7974006)*	*An. sinensis (KC797458)*	This study
hybridF_4_ x *sinensis*-2 = > (hybridF_5_-2)	SKSF5-2	*An. sinensis (KC797407)*	*An. sinensis (KC797459)*	This study
hybridF_4_ x *sinensis*-3 = > (hybridF_5_-3)	SKSF5-3	*An. sinensis (KC797408)*	*An. sinensis (KC797460)*	This study
hybridF_5_ x *sinensis*-1 = > (hybridF_6_-1)	SKSF6-1	*An. sinensis (KC797409)*	*An. sinensis (KC797461)*	This study
hybridF_5_ x *sinensis*-2 = > (hybridF_6_-2)	SKSF6-2	*An. sinensis (KC797410)*	*An. sinensis (KC797462)*	This study
hybridF_5_ x *sinensis*-3 = > (hybridF_6_-3)	SKSF6-3	*An. sinensis (KC797411)*	*An. sinensis (KC797463)*	This study
hybridF_6_ x *sinensis*-1 = > (hybridF_7_-1)	SKSF7-1	*An. sinensis (KC797412)*	*An. sinensis (KC797464)*	This study
hybridF_6_ x *sinensis*-2 = > (hybridF_7_-2)	SKSF7-2	*An. sinensis (KC797413)*	*An. sinensis (KC797465)*	This study
hybridF_6_ x *sinensis*-3 = > (hybridF_7_-3)	SKSF7-3	*An. sinensis (KC797414)*	*An. sinensis (KC797466)*	This study
hybridF_7_ x *sinensis*-1 = > (hybridF_8_-1)	SKSF8-1	*An. sinensis (KC797415)*	*An. sinensis (KC797467)*	This study
hybridF_7_ x *sinensis*-2 = > (hybridF_8_-2)	SKSF8-2	*An. sinensis (KC797416)*	*An. sinensis (KC797468)*	This study
hybridF_7_ x *sinensis*-3 = > (hybridF_8_-3)	SKSF8-3	*An. sinensis (KC797417)*	*An. sinensis (KC797469)*	This study
hybridF_8_ x *sinensis*-1 = > (hybridF_9_-1)	SKSF9-1	*An. sinensis (KC797418)*	*An. sinensis (KC797470)*	This study
hybridF_8_ x *sinensis*-2 = > (hybridF_9_-2)	SKSF9-2	*An. sinensis (KC797419)*	*An. sinensis (KC797471)*	This study
hybridF_8_ x *sinensis*-3 = > (hybridF_9_-3)	SKSF9-3	*An. sinensis (KC797420)*	*An. sinensis (KC797472)*	This study
hybridF_9_ x *sinensis*-1 = > (hybridF_10_-1)	SKSF10-1	*An. sinensis (KC797421)*	*An. sinensis (KC797473)*	This study
hybridF_9_ x *sinensis*-2 = > (hybridF_10_-2)	SKSF10-2	*An. sinensis (KC797422)*	*An. sinensis (KC797474)*	This study
hybridF_9_ x *sinensis*-3 = > (hybridF_10_-3)	SKSF10-3	*An. sinensis (KC797423)*	*An. sinensis (KC797475)*	This study
hybridF_10_ x *sinensis*-1 = > (hybridF_11_-1)	SKSF11-1	*An. sinensis (KC797424)*	*An. sinensis (KC797476)*	This study
hybridF_10_ x *sinensis*-2 = > (hybridF_11_-2)	SKSF11-2	*An. sinensis (KC797425)*	*An. sinensis (KC797477)*	This study
hybridF_10_ x *sinensis*-3 = > (hybridF_11_-3)	SKSF11-3	*An. sinensis (KC797426)*	*An. sinensis (KC797478)*	This study
hybridF_11_ x hybridF_11_-1	HF11-1	*An. sinensis (KC797427)*	*An. sinensis (KC797479)*	This study
hybridF_11_ x hybridF_11_-2	HF11-2	*An. sinensis (KC797428)*	*An. sinensis (KC797480)*	This study
hybridF_11_ x hybridF_11_-3	HF11-3	*An. sinensis (KC797429)*	*An. sinensis (KC797481)*	This study
hybridF_11_ x hybridF_11_-4	HF11-4	*An. sinensis (KC797430)*	*An. sinensis (KC797482)*	This study
Back crosses				
(*An. kleini* x *An. sinensis*) x *An. kleini*				
hybridF_1_ x *kleini*-1 = > (hybridF_2_-1)	KSKF2-1	*An. kleini (KC890843)*	*An. kleini (KC797483)*	This study
hybridF_1_ x *kleini*-2 = > (hybridF_2_-2)	KSKF2-2	*An. kleini (KC890844)*	*An. kleini (KC797484)*	This study
hybridF_1_ x *kleini*-3 = > (hybridF_2_-3)	KSKF2-3	*An. kleini (KC890845)*	*An. kleini (KC797485)*	This study
hybridF_2_ x *kleini-*1 = > (hybridF_3_-1)	KSKF3-1	*An. kleini (KC890846)*	*An. sinensis (KC797486)*	This study
hybridF_2_ x *kleini*-2 = > (hybridF_3_-2)	KSKF3-2	*An. kleini (KC890847)*	*An. sinensis (KC797487)*	This study
hybridF_2_ x *kleini*-3 = > (hybridF_3_-3)	KSKF3-3	*An. kleini (KC890848)*	*An. sinensis (KC797488)*	This study
hybridF_3_ x *kleini*-1 = > (hybridF_4_-1)	KSKF4-1	*An. kleini (KC890849)*	*An. sinensis (KC797489)*	This study
hybridF_3_ x *kleini*-2 = > (hybridF_4_-2)	KSKF4-2	*An. kleini (KC890850)*	*An. sinensis (KC797490)*	This study
hybridF_3_ x *kleini-*3 = > (hybridF_4_-3)	KSKF4-3	*An. kleini (KC890851)*	*An. sinensis (KC797491)*	This study
hybridF_4_ x *kleini*-1 = > (hybridF_5_-1)	KSKF5-1	*An. kleini (KC890852)*	*An. sinensis (KC797492)*	This study
hybridF_4_ x *kleini*-2 = > (hybridF_5_-2)	KSKF5-2	*An. kleini (KC890853)*	*An. sinensis (KC797493)*	This study
hybridF_4_ x *kleini*-3 = > (hybridF_5_-3)	KSKF5-3	*An. kleini (KC890854)*	*An. sinensis (KC797494)*	This study
*An. sinensis*	-	*An. sinensis (GU384700)*	-	[30]
		*-*	*An. sinensis (AY444351)*	[39]
*An. kleini*	*-*	*An. kleini (GU384713)*	*-*	[30]
		*-*	*An. kleini (GQ265917)*	[27]

### PCR and pyrosequencing for the detection of *An. sinensis* and *An. kleini* sequences

DNA was extracted from each adult female mosquito of *An. sinensis* and *An. kleini* using a NucleoSpin tissue kit (Macherey-Nagel GmbH and Co., Duren, Germany). The procedure for the pyrosequencing assay followed that previously described by [40]. The forward primer (Anop_COI_F: 5′-GAG CCC CTG ATA TAG CTT TTC CT-3′), and biotinylated reverse primers (Anop_COI_Rb: 5′- Biotin-CCA GAT GAA AGT GGG GGA TAA -3′), were designed to amplify a 142-bp fragment of COI, and a primer Anop_COI_S (5′-ATA AGT TTT TGA ATA TTA CC -3′) for pyrosequencing. Positive-control plasmids of each species were constructed by amplification of the 142-bp PCR products using Anop_COI_F and Anop_COI_R primers. They were ligated and transformed into a pGEM^®^-T Easy vector (Promega, WI) and an *Escherichia coli* JM109, respectively. The recombinant plasmids were sequenced bidirectionally in order to confirm the correction of data. The 142 bp was amplified from genomic DNA using the Anop_COI_F and Anop_COI_Rb primers. The reactions of PCR amplification were performed in a total volume of 25 μl containing 1X PCR buffer (Invitrogen, Carlsbad, CA) with 0.2 mM of each dNTP, 2 mM MgSO4, 0.4 μM of each primer, 0.625 U of Platinum *Taq* DNA polymerase high fidelity (Invitrogen, Carlsbad, CA) and 2 μl of the DNA sample. The PCR assay was conducted using a GeneAmp PCR system 9700 thermal cycler (Applied Biosystems, Singapore). The thermocycling program included: initial denaturation at 94°C for 5 min, with 40 cycles at 94°C for 30 sec, 57°C for 30 sec, and 72°C for 30 sec, and a final extension at 72°C for 7 min. PCR amplicons were detected by electrophoresis on 1.5% agarose gels. For pyrosequencing assays, 20 μl of the biotinylated PCR product of each sample was immobilized in the binding buffer with Streptavidin Sepharose™ beads (GE Healthcare BioSciences AB, Uppsala, Sweden). The beads together with the DNA were aspirated to a 96 format filter tool and passed through 70% ethanol and 0.2 M NaOH, and then washed with 10 mM Tris-acetate (pH 7.6) using a PyroMark™ Vacuum Prep Workstation (Biotage, Uppsala, Sweden). The beads were released subsequently into a PSQ™ 96 plate low (Biotage) containing 0.4 μM Anop_COI _S sequencing primer in the annealing buffer. The samples were heated to 80°C for 2 min before performing pyrosequencing reactions using PyroMark™ Gold Q96 SQA reagents and subjecting them to the PyroMark™ Q96 ID instrument (Biotage). Positive and negative controls were included in each pyrosequencing assay. Finally, the PyroMark™ Q96 ID software version 1.0 was used to analyze the results and generate a pyrogram.

## Results

### Hybridization experiments

**Figure 1:**
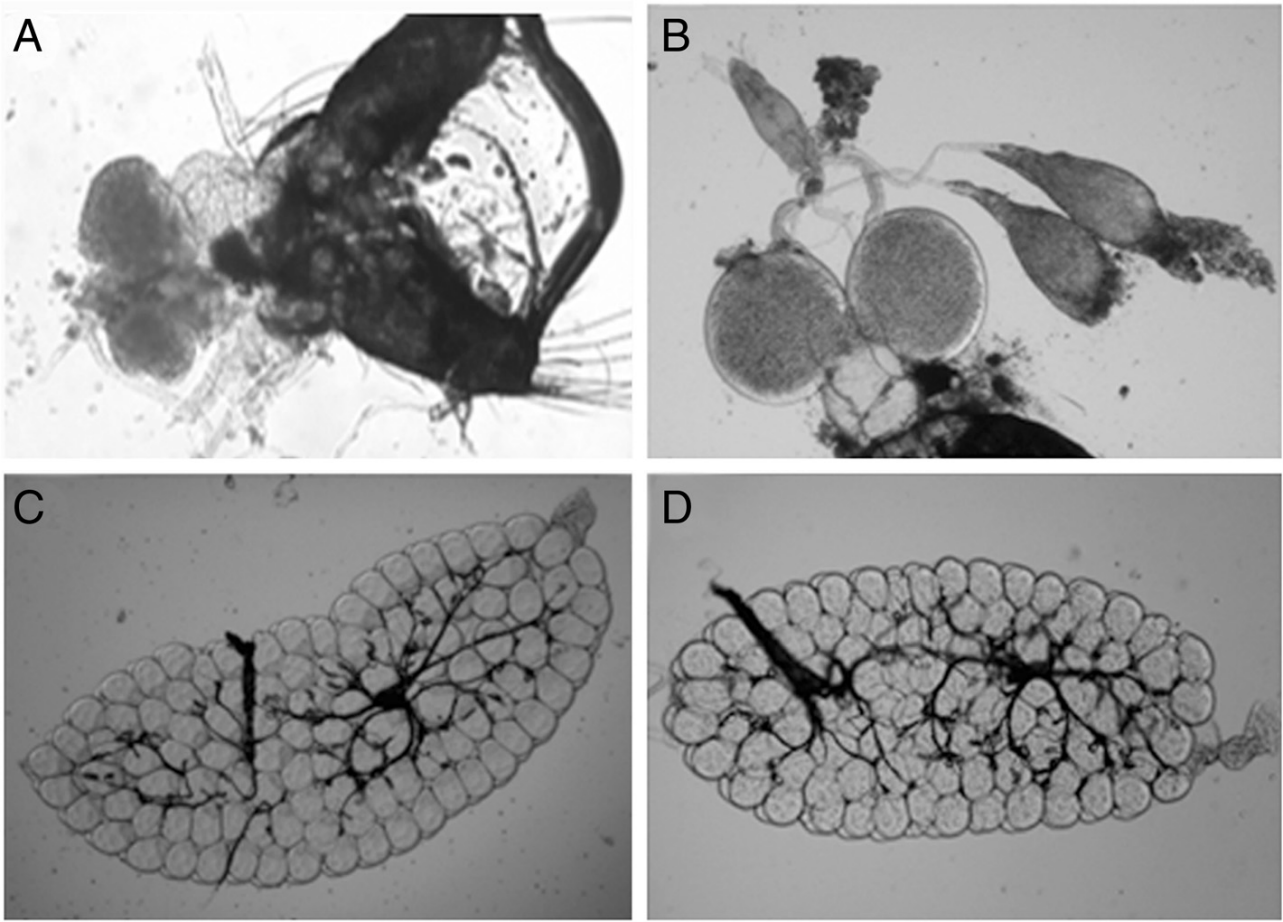
**Reproductive system of adult males and females. (A)** F_1_-hybrid adult male of *An. sinensis* x *An. kleini* showing atrophy of testes and accessory glands. **(B)** Backcross adult male of (*An. kleini* x *An. sinensis*)F_1_ x *An. kleini* showing normal development of testes and accessory glands. **(C)** F_1_-hybrid adult female of *An. sinensis* x *An. kleini* showing normal development of ovarian follicles. **(D)** F_1_-hybrid adult female of *An. kleini* x *An. sinensis* showing normal development of ovarian follicles.

The hatchability, pupation, emergence and adult sex-ratio of parental, reciprocal and back crosses, repeated backcrosses and hybrid crosses between *An. sinensis* and *An. kleini* were 88.06-92.57%, 77.96-92.97%, 94.10-96.98% and 0.81-0.89; 82.94-85.07%, 98.00-100%, 98.87-100% and 1.04-1.38; 61.10-69.01%, 100%, 97.10-100% and 1.13-1.43; 66.00-86.61%, 87.87-100%, 90.07-100% and 0.96-1.38, and 95.14%, 91.05%, 84.91% and 0.94; respectively (Table 2). All crosses yielded viable progenies, with no evidence of genetic incompatibility being observed among them, except for only sterile F_1_-progeny males of which the atrophy of testes and accessory glands were recovered from the reciprocal crosses in both directions (*An. sinensis* × *An. kleini* and *An. kleini* x *An. sinensis*) (Figure 1A), while normal development occurred in all males from repeated backcrosses (Figure 1B). On the other hand, all females from F_1_-hybrids and repeated backcrosses yielded normal development of ovarian follicles (Figures 1C and D). Regarding repeated backcross groups, the experiments of (*An. sinensis* x *An. kleini*) × *An. sinensis* were carried out from F_1-10_, whereas those of (*An. kleini* × *An. sinensis*) × *An. kleini* were investigated from F_1-5_. The reason for this was that the repeated backcross of (*An. kleini* × *An. sinensis*) × *An. kleini* could be carried out in only the fifth generation, which led to a lack of hybrids for further experimentation (repeated twice). Regarding hybrid crosses, the experiment was performed only on the F_11_ of [(*An. sinensis* × *An. kleini*) × *An. sinensis*] × [(*An. sinensis* × *An. kleini*) × *An. sinensis*].

**Table 2:** **Hybridization experiments between isolines of *An. sinensis *** **and *An. kleini***

**Crosses (Female x Male)**	Total eggs (number)^*^	**Embryonation rate**^**†**^	**Hatched n (%)**	**Pupation n (%)**	**Emergence n (%)**	**Total emergence n (%)**	
						**Female**	**Male**
Parental crosses							
*An. sinensis* x *An. sinensis*	538 (258, 280)	96	498 (92.57)	463 (92.97)	449 (96.98)	211 (46.99)	238 (53.01)
*An. kleini* x *An. kleini*	469 (263, 206)	89	413 (88.06)	322 (77.96)	303 (94.10)	136 (44.88)	167 (55.12)
Reciprocal crosses							
*An. sinensis* x *An. kleini*	529 (288, 241)	86	450 (85.07)	441 (98.00)	436 (98.87)	253 (58.03)	183 (41.97)^**††**^
*An. kleini* x *An. sinensis*	422 (239, 183)	83	350 (82.94)	350 (100.00)	350 (100.00)	178 (50.86)	172 (49.14)^**††**^
Back crosses							
(*An. sinensis* x *An. kleini*)F_1_ x *An. sinensis*	401 (218, 183)	63	245 (61.10)	245 (100.00)	238 (97.10)	140 (58.82)	98 (41.18)
(*An. sinensis* x *An. kleini*)F_2_ x *An. sinensis*	397 (207, 190)	88	332 (83.67)	309 (93.07)	309 (100.00)	173 (55.96)	136 (44.04)
(*An. sinensis* x *An. kleini*)F_3_ x *An. sinensis*	386 (211, 175)	74	282 (73.05)	265 (93.97)	265 (100.00)	143 (53.96)	122 (46.04)
(*An. sinensis* x *An. kleini*)F_4_ x *An. sinensis*	413 (240, 173)	79	319 (77.24)	284 (89.02)	259 (91.20)	137 (52.90)	122 (47.10)
(*An. sinensis* x *An. kleini*)F_5_ x *An. sinensis*	412 (232, 180)	69	282 (68.45)	282 (100.00)	254 (90.07)	139 (54.72)	115 (45.28)
(*An. sinensis* x *An. kleini*)F_6_ x *An. sinensis*	409 (226, 183)	86	318 (77.75)	308 (96.86)	295 (95.78)	171 (57.97)	124 (42.03)
(*An. sinensis* x *An. kleini*)F_7_ x *An. sinensis*	365 (147, 218)	87	307 (84.11)	301 (98.05)	295 (98.01)	157 (53.22)	138 (46.78)
(*An. sinensis* x *An. kleini*)F_8_ x *An. sinensis*	355 (154, 201)	84	284 (80.00)	281 (98.94)	267 (95.02)	139 (52.06)	128 (47.94)
(*An. sinensis* x *An. kleini*)F_9_ x *An. sinensis*	315 (190, 125)	89	271 (86.03)	271 (100.00)	271 (100.00)	136 (50.18)	135 (49.82)
(*An. sinensis* x *An. kleini*)F_10_ x *An. sinensis*	336 (162, 174)	94	291 (86.61)	266 (91.40)	257 (96.62)	126 (49.03)	131 (50.97)
(*An. kleini* x *An. sinensis*)F_1_ x *An. kleini*	497 (239, 258)	74	343 (69.01)	343 (100.00)	343 (100.00)	182 (53.06)	161 (46.94)
(*An. kleini* x *An. sinensis*)F_2_ x *An. kleini*	427 (226, 201)	77	305 (71.43)	268 (87.87)	268 (100.00)	147 (54.85)	121 (45.15)
(*An. kleini* x *An. sinensis*)F_3_ x *An. kleini*	421 (209, 212)	70	286 (67.93)	257 (89.86)	257 (100.00)	126 (49.03)	131 (50.97)
(*An. kleini* x *An. sinensis*)F_4_ x *An. kleini*	458 (211, 247)	79	328 (66.00)	302 (92.07)	287 (95.03)	155 (54.01)	132 (45.99)
F_11_ hybrid crosses							
[(*An. sinensis* x *An. kleini*)F_11_ x *An. sinensis*]	329 (171, 158)	96	313 (95.14)	285 (91.05)	242 (84.91)	117 (48.35)	125 (51.65)
x [(*An. sinensis* x *An. kleini*)F_11_ x *An. sinensis*]							

*Two selective egg-batches of inseminated females from each cross; ^**†**^Dissection from 100 eggs; n = number.

^**††**^Sterile male hybrids with atrophy testes and accessory glands.

### PCR species identification

For molecular identification, sequences of nuclear ITS2 and mitochondrial COI genes were determined for both the parent mosquitoes and their hybrid progenies. Sequences of *An. sinensis* parents (F_0_) were well matched in both genes with those in the GenBank nucleotide sequence database. The four specimens of an *An. kleini* parent (F_0_) were also checked, and they all matched *An. kleini* based on ITS2 sequences. However, their COI gene, *An. kleini* matched in both species, with 1 being *An. kleini* and 3 *An. sinensis* (Table 1).

The F_1_-hybrid progenies have mixed sequences in their nuclear ITS2 gene. They have heterogeneous ITS2 sequences because they receive ribosomal RNA genes, including ITS2 from both parents. As for a maternal, mitochondrial COI gene, the hybrids followed the trait of the mother. All the progenies of *An. sinensis* × *An. kleini* matched *An. sinensis* based on COI sequences. However, progenies of *An. kleini* × *An. sinensis* matched both *An. kleini* and *An. sinensis* based on COI sequences, in which two progenies matched *An. sinensis* and one *An. kleini* (Table 1).

Two backcrossing groups were checked, with one being a progeny of (*An. sinensis* × *An. kleini*) × *An. sinensis* and the other a progeny of (*An. kleini* × *An. sinensis*) × *An. kleini*. In the (*An. sinensis* × *An. kleini*) × *An. sinensis* back crossing group, all the progenies were well matched *An. sinensis* NCBI sequences in the ITS2 sequence, except for 3 of F_2_-hybrid progenies (mixed). Also, all sequences of the COI gene showed *An. sinensis*. In the (*An. kleini* × *An. sinensis*) × *An. kleini* backcrossing group, all sequences of the ITS2 showed *An. kleini*, but sequences of the COI gene matched *An. kleini* only in 3 of F_2_-hybrid progenies, and the remaining F_3-5_ progenies matched *An. sinensis* (Table 1).

### Phylogenetic analysis

**Figure 2:**
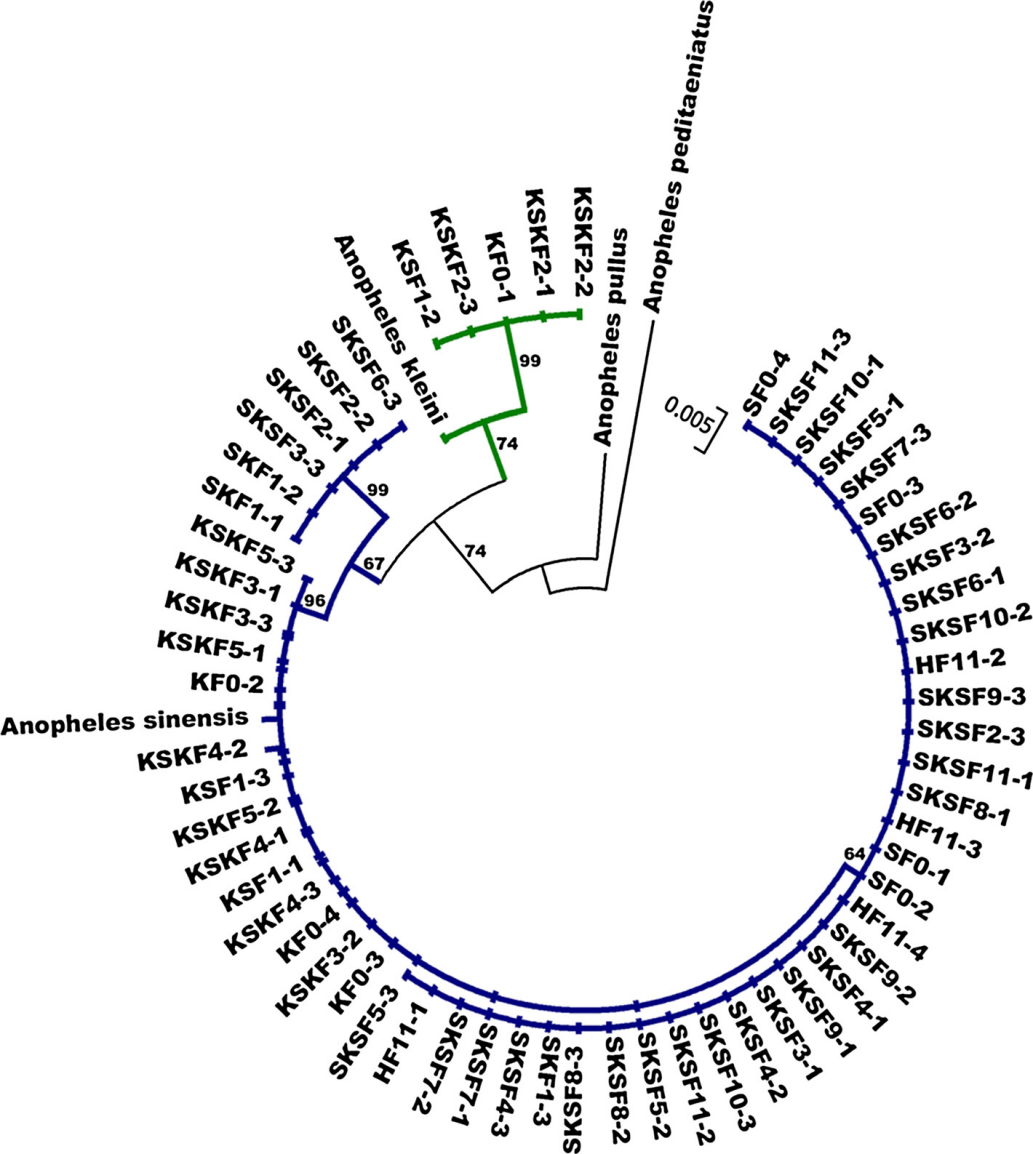
**Neighbor-joining tree of *An. sinensis *and *An. kleini *based on COI sequences.** Numbers on branches are bootstrap values (%) of NJ analysis. Only greater than 50% bootstrap values are shown. Bars represent 0.005 substitutions per site. Detailed code of samples is shown in Table 1.

The neighbor-joining (NJ) tree was constructed based on COI sequences in order to determine sequence divergence among the species examined (Table 1, Figure 2). The NJ tree showed concordant results with the PCR assay in all samples, which were divided into two major clades with 67-74% bootstrap support. Clade I consisted of 55 samples of *An. sinensis*. It is interesting to note that among the 55 samples, 12 (KF0-2, KF0-3, KF0-4, KSKF3-1, KSKF3-2, KSKF3-3, KSKF4-1, KSKF4-2, KSKF4-3, KSKF5-1, KSKF5-2 and KSKF5-3) and 8 (SKF1-1, SKF1-2, SKF1-3, KSF1-1, KSF1-3, SKSF2-1, SKSF2-2 and SKSF2-3), were identified as *An. kleini* and mixed sequences of both species, respectively, based on ITS2 sequences. Furthermore, these samples were placed within the same clade as the published sequence of *An. sinensis* (mean genetic distances = 0.003). Clade II comprised 5 samples of *An. kleini* derived from parental (KF0-1), reciprocal (KSF1-2) and backcrosses (KSKF2-1, KSKF2-2 and KSKF2-3) based on COI sequences (Table 1). The mean genetic distance between these two species was 0.023.

### Pyrosequencing analysis

**Figure 3:**
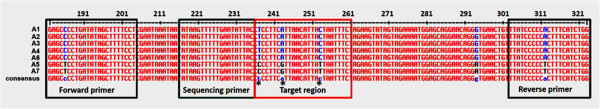
**Alignment of the cytochrome *c *oxidase subunit I (COI) gene derived from seven samples.** Position of the forward primer (Anop_COI_F) and biotinylated reverse primer (Anop_COI_Rb) for template amplification are shown in the black boxes, while the sequencing primer (Anop_COI_S) and target region are shown in the green and red boxes, respectively. Asterisks indicate position of the target region used for species level identification.

**Figure 4:**
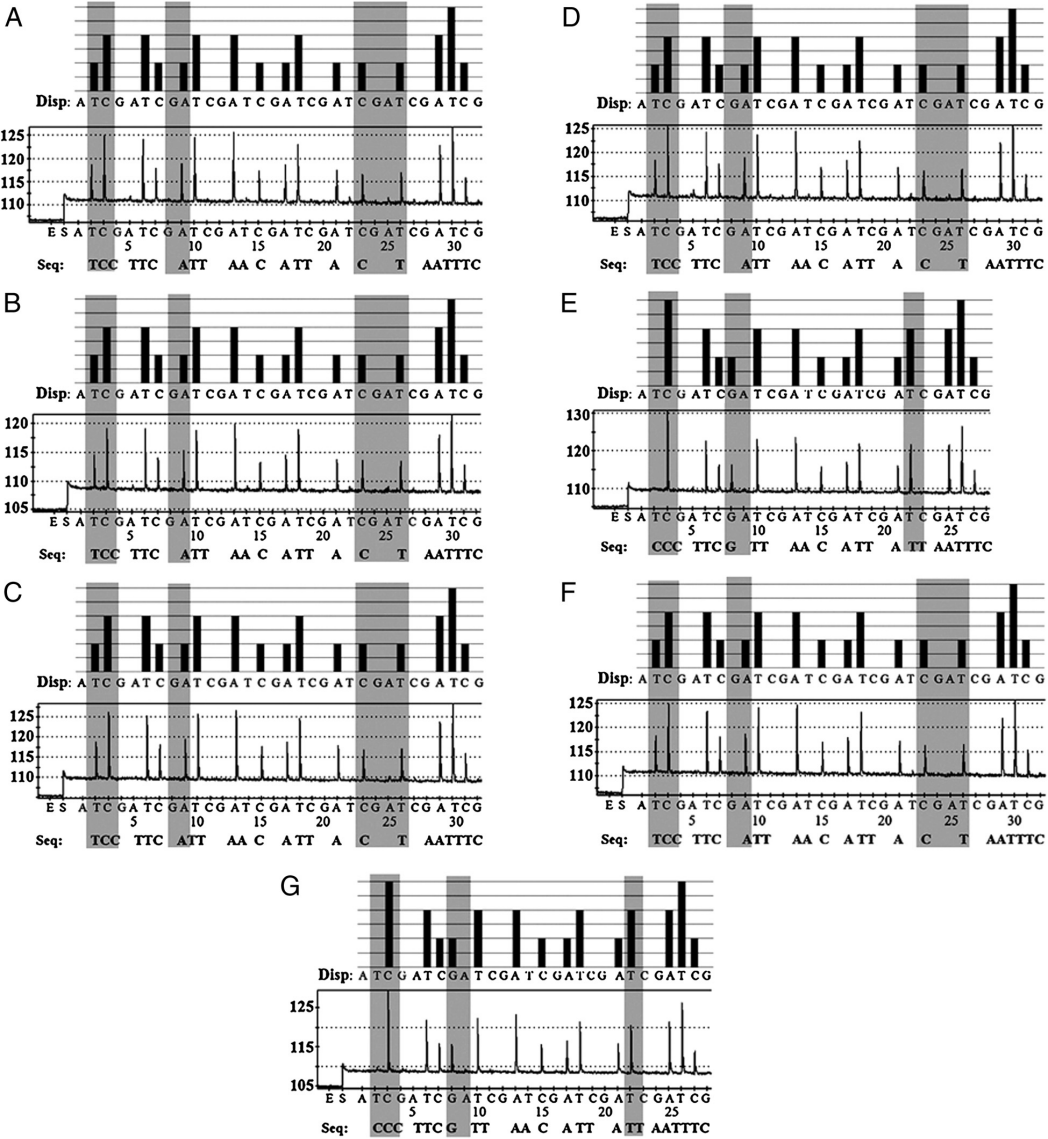
**Pyrograms showing sequence analysis (SQA) of 24-base fragments of the COI gene.** Group 1; **(A)** Parental: *An. sinensis* F_0_, **(B)** F_1_: *An. sinensis* x *An. kleini* -- > hybrid F_1_, **(C)** F_5_: hybrid F_4_ x *An. sinensis* -- > hybrid F_5_, **(D)** F_10_: hybrid F_9_ x *An. sinensis* -- > hybrid F_10_, and **(F)** F_1_: *An. kleini* x *An. sinensis* -- > hybrid F_1_. Group 2; **(E)** Parental: *An. kleini* F_0_ and **(G)** F_5_: hybrids F_4_ x *An. kleini* -- > hybrid F_5_ using pyrosequencing. Theoretical pyrogram patterns (top of each panel) and representative raw data (bottom of each panel) of control DNA extracted from each *An. sinensis* and *An. kleini* by pyrosequencing are shown. Pyrosequencing was performed by addition of enzyme (E), substrate (S), and four different nucleotides. The letters under the black bars show the dispensation (Disp:) order. The actual sequence detected by pyrosequencing is indicated below the panels after “Seq”. The Y-axis represents the level of fluorescence emitted by incorporating a nucleotide base, and the X-axis represents the total number of bases added at that point in time; A, T, C, G nucleotide bases. The light gray areas show the pyrogram for identifying each of the two groups of *An. sinensis* and *An. kleini.*

The 24-nucleotide target region of the COI gene, including positions 237-260 of *An. sinensis* and *An. kleini*, was useful in classifying the sequenced species into 2 groups, as shown in Table 3 and Figures 3 and 4. The first group consisted of Parental: *An. sinensis* F_0_ (Figure 4A), F_1_: *An. sinensis* x *An. kleini* -- > hybrid F_1_ (Figure 4B), F_5_: hybrid F_4_ x *An. sinensis* -- > hybrid F_5_ (Figure 4C), F_10_: hybrid F_9_ x *An. sinensis* -- > hybrid F_10_ (Figure 4D), and F_1_: *An. kleini* x *An. sinensis* -- > hybrid F_1_ (Figure 4F) and another group comprised Parental: *An. kleini* F_0_ (Figure 4E) and F_5_: hybrid F_4_ x *An. kleini* -- > hybrid F_5_ (Figure 4G). Both groups differed from each other in three nucleotide positions (T237C, A243G and C253T) (Table 3). The positive-control plasmids showed similar results to the seven samples in the pyrogram, whereas, a negative control did not provide the pyrogram result.

**Table 3:** **Sample details and nucleotide positions used for discriminating between *An. sinensis *** **and *An. kleini *** **based on COI sequences**

**Code no.**	**Samples**	**Nucleotide at position**		
	**(Female x Male)**	**237**	**243**	**253**
A1^*^	Parental: *An. sinensis* F_0_	T	A	C
A2^*^	F_1_: *An. sinensis* x *An. kleini* -- > hybrid F_1_	T	A	C
A3^*^	F_5_: hybrid F_4_ x *An. sinensis* -- > hybrid F_5_	T	A	C
A4^*^	F_10_: hybrid F_9_ x *An. sinensis* -- > hybrid F_10_	T	A	C
A5^**†**^	Parental: *An. kleini* F_0_	C	G	T
A6^*^	F_1_: *An. kleini* x *An. sinensis* -- > hybrid F_1_	T	A	C
A7^**†**^	F_5_: hybrid F_4_ x *An. kleini* -- > hybrid F_5_	C	G	T

^*^group 1, COI sequence matched with *An. sinensis*.

^**†**^group 2, COI sequence matched with *An. kleini*.

## Discussion

Introgression or introgressive hybridization is the movement of a gene (gene flow) between species through hybridization by repeated backcrossing of an interspecific hybrid with one of its parent species. It can have important effects on dynamics of the hybrid zone, speciation and adaptive radiation [41]. The variation of mitochondrial DNA is important and used widely for indirect studies of gene flow [42]. Thelwell *et al*. [43] reported evidence of mitochondrial (ND5) introgression between *An. bwambae* and *An. gambiae.* Consequently, extensive investigations of introgression between *An. gambiae* and *An. arabiensis*, and *An. bwambae* and *An. gambiae* have been documented systematically and extensively during the past decade [44-46]. Additionally, Walton *et al*. [42] demonstrated that mitochondrial DNA introgressed from *An. baimaii* (= *dirus* D) into *An. dirus* (= *dirus* A), and Morgan *et al*. [47] reported evidence to support the mitochondrial introgression between *An. baimaii* and *An. dirus* by the high levels of bidirectional mitochondrial gene flow detected between these 2 species. A large number of anopheline species were reported to be capable of interspecific hybridizations under laboratory conditions [48]. However, few species have succeeded in natural hybridization, for example, between *An. gambiae* and *An. arabiensis*[23], *An. bwambae* and *An. gambiae*[43], *An. scanloni* (= *dirus* C) and *An. baimaii* (= *dirus* D) [24], and *An. minimus* and *An. harrisoni*[28,29]. Recent discovery of the natural hybrid between *An. sinensis* and *An. kleini*[27], and successful establishment of iso-female line colonies of these 2 anopheline species has urged this study to form a reliable systematic procedure to confirm this natural event and/or perform an introgressive study. The results of sterile F_1_-hybrid adult males with atrophy of accessory glands and testes obtained from the reciprocal crosses between these 2 anopheline species are in keeping with “Haldane Rule”, which states that in interspecific crosses, the heterogametic sex (X, Y) will show sterility or viability problems before the homogametic sex [49]. Thus, the results of this study agree with those of Davidson [50], who reported that female and male hybrids obtained from *An. gambiae* and *An. arabiensis* were fertile and sterile, respectively. Furthermore, the results of this study are in accordance with crossing studies in the laboratory by Baimai *et al*. [51]. They demonstrated that F_1_ hybrids obtained from the cross of female *An. dirus* with male *An. scanloni* were fertile and viable, with an exception of sterile males. It was interesting to note that the repeated backcross progenies in both directions, which resulted in obtaining *An. sinensis* from hybrids of F_2_-_11_ progenies [(*An. sinensis* x *An. kleini*) x *An. sinensis*] and F_3_-_5_ progenies [(*An. kleini* x *An. sinensis*) x *An. kleini*], indicated the presence of introgressive hybridization between *An. sinensis* and *An. kleini*.

Regarding PCR identification of parental specimens, the exact species of *An. sinensis* and *An. kleini* were used in this study based on both the nuclear ITS2 and mitochondrial COI genes. Interestingly, 4 iso-female lines of *An. kleini* showed the correct gene trait in the ITS2, but their COI sequences matched both species (i.e., *An. kleini*: 1 iso-female line, and *An. sinensis*: 3 iso-female lines). Therefore, the authors assumed that the COI sequences of *An. kleini* had been replaced by those of *An. sinensis*. Subsequently, reciprocal and repeated backcrosses were performed to clarify our hypothesis. Most COI sequence results of the progenies obtained from reciprocal and repeated backcrosses revealed that the mitochondrial COI gene introgressed from *An. sinensis* into *An. kleini*. This event resulted from introgression that occurred between these two species via consecutive repeated backcrosses. Thus, the sample identification of these 2 wild-caught species should be careful only when the COI barcoding region has been applied. Also, results from phylogenetic analysis confirmed the existence of an introgression phenomenon between them. Furthermore, the results from this study are in agreement with those of Petit and Excoffier [52]. They suggested that in species with male-biased dispersal (heterogametic sex), mtDNA markers should introgress more readily than nuclear ones.

Pyrosequencing is a unique sequencing method that was developed as an alternative to classical DNA sequencing for short- to medium-read applications. It is an accurate, simple and flexible bioluminometric method, which does not need labeled nucleotides or gel electrophoresis [53]. To date, this technology has been used successfully for high throughput identification of bacteria [54,55], virus [56,57], protozoan parasites [53,58-60] and helminthes [40]. This study applied the pyrosequencing technology for reliable identification from seven samples of *An. sinensis* and *An. kleini*. The results were consistent with those of dideoxy sequencing and phylogentic analysis in six samples, except for the one (sample code no. A7) matched with *An. kleini* COI sequences. This sample possibly had a different mitochondrial haplotype from other hybrid progenies. More recently, the next-generation sequencing provided a good explanation of interspecific gene flow between *An. gambiae* and *An. arabiensis*[61] and *An. gambiae* M and S [62,63].

The effectiveness of a vector control strategy, and genetically modified strains of mosquitoes in a population that is unable to transmit malarial parasites, relies upon the gene flow within species and introgression [64]. Remarkably, Rheindt and Edwards [65] mentioned concern that in the long-term introgression with newcomer species may lead to a loss of genetic integrity in native species. Detection of natural hybridization is rare but meaningful in terms of horizontal transfer of advantageous genes, such as those in malaria susceptibility, particularly when species that allow hybridization are susceptible or refractory to malarial parasites [9,66,67]. This event is also involved in insecticide resistant genes, e.g., the *kdr* gene [68,69] and *ace-1* gene [70]. In addition, Morgan *et al*. [71] stated that the absence or presence of gene flow between populations and species has an impact on the dynamics of malaria transmission as well as construction of effective strategies for controlling malaria vectors. Our studies presented the introgressive events through consecutive repeated backcrosses under laboratory conditions, in which the mtDNA gene could be moved from one species to another. However, natural movement of the refractory gene to vivax malaria between sympatric populations of a low potential vector (*An. sinensis*) and a high potential vector (*An. kleini*) needed intensive and systematic clarification. This new body of knowledge is anticipated to elucidate the promising strategies for replacing populations of high potential vectors with that of low potential vectors by using genetic manipulation through the gene introgression mechanism.

## Conclusions

A single gravid, natural hybrid female between high (*An. kleini*) and low (*An. sinensis*) potential vectors of *P. vivax* was discovered in Paju City, Republic of Korea (ROK). The discovery of natural hybrids between these two anopheline species has led to systematic investigations of various aspects that clarify this event. Hybridization experiments used iso-female line colonies of these anophelines together with DNA analysis of ribosomal DNA [second internal transcribed spacer (ITS2)] and mitochondrial DNA [cytochrome *c* oxidase subunit I (COI)] of the parental colonies, F_1_-hybrids and repeated backcross progenies were performed intensively by using a PCR-based assay and pyrosequencing technology. The results revealed that introgression of the COI gene between *An. sinensis* and *An. kleini* was involved in this phenomenon. The pure *An. sinensis* obtained from hybrids of repeated backcross progenies in both directions, i.e., F_2-11_ progeny [(*An. sinensis* x *An. kleini*) x *An. sinensis*] and F_3**-**5_ progeny [(*An. kleini* x *An. sinensis*) x *An. kleini*] provided obvious supportive evidence. The results emphasize a promising way to replace the population of a high potential vector (*An. kleini*) with that of a low potential vector (*An. sinensis*) through the mechanism of gene introgression.

## Competing interests

The authors declare that they have no competing interests.

## Authors’ contributions

All the authors contributed significantly to this study. WC and GSM designed the experiments, carried out field and laboratory experiments, interpreted the results, and wrote the manuscript. AS participated in hybridization experiments and molecular identifications. PMI, CT and VL carried out pyrosequencing analysis. All the authors read and approved the final version of the manuscript.
